# Evaluating Safety and Anatomical Eligibility for Paranasal Implants in the Atrophic Maxilla: A Segmentation-Assisted Proof-of-Concept Study

**DOI:** 10.3390/jcm15124750

**Published:** 2026-06-18

**Authors:** Andra Patricia David, Silviu Brad, Laura-Cristina Rusu, Ovidiu Tiberiu David, Andra Ardelean, Robert-Angelo Tuce, Marius Traian Leretter

**Affiliations:** 1 “Victor Babes” University of Medicine and Pharmacy Timisoara, 2 Eftimie Murgu Sq., 300041 Timisoara, Romania; andra.david@umft.ro (A.P.D.); ardelean.andra@umft.ro (A.A.); 2 Department of Radiology, “Victor Babes” University of Medicine and Pharmacy Timisoara, 2 Eftimie Murgu Sq., 300041 Timisoara, Romania; brad.silviu@umft.ro; 3 Department of Oral Pathology, Multidisciplinary Center for Research, Evaluation, Diagnosis and Therapies in Oral Medicine, “Victor Babes” University of Medicine and Pharmacy Timisoara, 2 Eftimie Murgu Sq., 300041 Timisoara, Romania; 4 Faculty of Physics, West University of Timisoara, 4 Vasile Parvan Blvd., 300223 Timisoara, Romania; 5 Department of Functional Sciences, Multidisciplinary Center for Research, Evaluation, Diagnosis and Therapies in Oral Medicine, “Victor Babes” University of Medicine and Pharmacy Timisoara, 2 Eftimie Murgu Sq., 300041 Timisoara, Romania; 6 Radiologie CBCT SRL, Iulius Mall, 2 Consiliul Europei Sq., 300627 Timisoara, Romania; 7 Department of Functional Sciences, “Victor Babes” University of Medicine and Pharmacy Timisoara, 2 Eftimie Murgu Sq., 300041 Timisoara, Romania; tuce.robert@gmail.com; 8 Department of Prosthodontics, Multidisciplinary Center for Research, Evaluation, Diagnosis and Therapies in Oral Medicine, “Victor Babes” University of Medicine and Pharmacy Timisoara, 2 Eftimie Murgu Sq., 300041 Timisoara, Romania; leretter.marius@umft.ro

**Keywords:** paranasal implants, atrophic maxilla, cone-beam computed tomography, image segmentation, surgical planning, computer-assisted, nasolacrimal duct, dental implantation, treatment eligibility, cortical bone, three-dimensional imaging

## Abstract

**Background/Objectives**: Implant placement in transnasal and paranasal regions of the severely atrophic maxilla is challenged by complex anatomy and proximity to critical structures, particularly the nasolacrimal duct (NLD). While cortical anchorage is considered important for implant stability, structured methods for evaluating anatomical eligibility and anatomical risk during planning remain limited. This proof-of-concept study aimed to describe a segmentation-assisted workflow for anatomical assessment of potential paranasal implant trajectories. **Methods**: A single-case proof-of-concept workflow was developed using CBCT imaging and multi-component anatomical bone segmentation (MCABS). Segmented anatomical structures were used to selectively visualize cortical pathways within the anterior maxilla. Implant planning was performed using axial, non-tilted trajectories. Particular attention was directed toward visualization of the spatial relationship between the planned implant pathway and the nasolacrimal duct. Workflow feasibility was further explored through study-model fabrication, guided implant insertion, and axis-based verification. **Results**: The proposed workflow enabled selective visualization of cortical structures and facilitated identification of anatomically favorable implant trajectories within the paranasal region. The relationship between the planned implant pathway and the nasolacrimal duct could be directly assessed using the segmented anatomical model. Guided insertion in the study model demonstrated concordance between planned and executed implant axes, supporting the technical feasibility of the workflow. **Conclusions**: Within the limitations of a single-case proof-of-concept study, the proposed segmentation-assisted workflow may contribute to preoperative anatomical assessment of potential paranasal implant trajectories and their relationship to adjacent anatomical structures. The workflow should be regarded as a methodological demonstration rather than a validated clinical protocol. Further anatomical, reproducibility, biomechanical, and clinical studies are required before broader clinical adoption can be considered.

## 1. Introduction

The rehabilitation of the severely atrophic maxilla remains one of the most challenging situations in implant dentistry, largely due to the heterogeneous ways edentulous maxillae are defined in the literature [1]. As a result, patient eligibility for paranasal implants should rely on an anatomy-driven assessment rather than fixed classifications, focusing on the degree of atrophy and the availability of cortical anchorage in the nasal and paranasal regions. Progressive alveolar bone resorption, frequently associated with maxillary sinus pneumatization, may severely reduce the amount and distribution of residual bone available for implant placement, particularly in the anterior maxilla. Although bone grafting procedures have traditionally been used to overcome these limitations, they are associated with higher costs, longer treatment times, and limited predictability, especially when immediate loading is intended [2,3,4]. In response to these limitations, graftless treatment concepts have progressively evolved toward the use of remote cortical anchorage sites, including the zygomatic bone, pterygomaxillary region, and anatomically strategic maxillary buttresses [4,5]. This shift reflects a broader change in perspective: treatment planning in the atrophic maxilla can no longer rely exclusively on the overall volume of remaining bone but must increasingly consider the distribution, accessibility, and mechanical relevance of cortical bone structures. From this standpoint, cortical bone plays a central role in implant stability and long-term function. Anatomical structures such as the lateral nasal wall, piriform rim, frontal process of the maxilla, and the medial wall of the maxillary sinus may provide clinically relevant anchorage zones in patients with severe maxillary atrophy [6,7]. Recent anatomical and clinical studies have further emphasized that cortical engagement is particularly relevant for achieving primary stability and facilitating immediate loading in anatomically compromised maxillae [8,9]. However, conventional imaging-based planning often treats the maxilla as a unified osseous volume, without sufficiently distinguishing between cortical and trabecular compartments. From a functional and biomechanical perspective, this simplification may be limiting. Cortical bone provides the principal structural support for implant anchorage, whereas trabecular bone contributes differently and may obscure the visualization of mechanically favorable pathways when both are interpreted together as a single undifferentiated mass [7,10]. For this reason, a more selective and anatomically resolved analysis of the maxilla may be advantageous, particularly in regions where cortical support is sparse, discontinuous, or geometrically complex. Accurate segmentation of jawbone structures from CBCT images represents a fundamental step in digital implant planning, particularly when differentiating between cortical and trabecular bone compartments. These two components exhibit distinct structural and biomechanical properties, both of which are directly related to primary implant stability and treatment outcomes. However, reliable segmentation of cortical and trabecular bone remains challenging in CBCT imaging due to inherent limitations such as low contrast, noise, and variability in gray values across devices. Traditional segmentation approaches, including global thresholding and region-growing techniques, often fail to accurately capture complex internal bone structures or to distinguish between cortical and trabecular compartments. More advanced methods, including adaptive thresholding, semi-automated protocols, and deep learning-based segmentation systems, have been proposed to address these limitations and improve accuracy, reproducibility, and efficiency in jawbone analysis. Medical image segmentation can currently be performed using a broad range of software solutions, encompassing fully automatic, automatic, manual, and semi-automatic workflows, depending on the extent of user interaction required. Among these, ITK-SNAP is a well-established tool for manual and user-guided semi-automatic segmentation, 3D Slicer provides an open-source interactive platform for 3D medical image segmentation, and Mimics has been widely used for computer-aided anatomical analysis and semi-automated processing [11,12,13]. We selected these three software platforms as they are representative of the current landscape in segmentation workflows. However, none of them incorporates a direct integration with a CBCT device, nor are they specifically designed as dedicated dental software solutions. At the same time, the literature indicates that fully automatic approaches, although increasingly sophisticated, may still encounter limitations in anatomically complex regions and often require user-guided correction or refinement. For this reason, semi-automatic and dedicated task-specific solutions remain highly relevant in clinical practice. In the present study, we used the DAVIS Toolkit for Segmentation (New 3D AI), Eyes of AI Pty Ltd. (Sydney, Australia). This software solution is specifically developed for multi-component anatomical bone segmentation (MCABS), featuring automated segmentation that enables efficient and reproducible identification of distinct anatomical bone structures with minimal manual input [14,15]. Its dedicated design for detailed osseous segmentation, together with its integration within the ecosystem of the CBCT manufacturer VATECH, Republic of Korea, supports its relevance for dentomaxillofacial applications and its potential value in advanced anatomical planning workflows. Within this evolving field, transnasal and perinasal implant strategies have emerged as valuable alternatives or adjuncts to more established graftless solutions. These approaches make use of residual bone located between the nasal cavity and the maxillary sinus, as well as bone within the frontal process of the maxilla and adjacent perinasal structures, to obtain anterior anchorage in cases where conventional implants are not feasible [16]. Clinical reports and case series have suggested that such implants may reduce anterior cantilever, improve anteroposterior implant spread, and complement zygomatic-based rehabilitation concepts in full-arch treatment of the severely atrophic maxilla [8,17,18,19,20]. This is particularly relevant in the context of full-arch rehabilitation concepts in which implant distribution and biomechanics are critical. In cases where standard all-on-4 configurations become limited by severe anterior and posterior atrophy, transnasal or perinasal anchorage may represent an additional structural resource capable of supporting more favorable implant positioning and reducing prosthetic compromise [6,8,17]. In this sense, these approaches should not be understood only as isolated surgical techniques but also as part of a broader anatomical strategy for improving cortical support in complex maxillary reconstructions. In severe maxillary atrophy, full-arch rehabilitation depends not only on posterior implant anchorage but also on the presence of reliable anterior support [21]. Existing concepts such as All-on-4 and its M-4 and V-4 variations have shown that the anterior maxilla plays an important biomechanical role by contributing to implant distribution, anteroposterior spread, and cantilever reduction. In these approaches, anterior support is typically obtained through tilted implant trajectories directed toward dense cortical structures in the nasal or midline region [22]. This principle remains relevant in extended rehabilitation strategies, including those performed in combination with zygomatic and pterygoid implants, where maintenance of a coherent anterior–posterior anchorage framework is essential [23,24,25,26,27,28]. The biomechanical rationale for such strategies is closely related to the concept of bicortical anchorage. Bicortical engagement is widely recognized as an important factor for achieving enhanced primary stability, particularly in immediate loading protocols and in low-density maxillary bone [10,28]. In anatomically favorable perinasal situations, however, the implant axis may potentially pass through a more complex cortical configuration [29]. Rather than engaging only two cortices, certain trajectories may intersect a region that includes the lateral nasal wall cortex, the vestibular maxillary cortex, and the medial sinus wall. This possible tricortical relationship has not yet been systematically explored as a segmentation-based planning concept, but it may have important implications for implant trajectory design and for the interpretation of structural support in the atrophic maxilla. Primary implant stability is strongly influenced by the degree of cortical bone engagement, since the mechanical fixation achieved at implant insertion depends not only on bone quantity and density but also on the thickness and strategic distribution of cortical bone at the recipient site. Available evidence suggests that thicker cortical bone is associated with improved mechanical stability, commonly expressed by higher insertion torque and more favorable implant stability parameters. Moreover, bicortical anchorage has been reported to provide superior primary stability compared with monocortical support, indicating that engagement of more than one cortical layer may substantially improve the mechanical behavior of the implant at placement. This is particularly relevant in demanding anatomical situations and in treatment concepts that require high primary stability for immediate or early loading [30,31]. Although direct clinical evidence on tricortical anchorage remains limited, it is reasonable to infer that the engagement of multiple cortical boundaries may further strengthen implant fixation by increasing resistance to micromotion and promoting a more favorable distribution of functional forces. Therefore, the three-dimensional identification of cortical, bicortical, or even tricortical anchorage pathways may represent an important objective in advanced implant planning [7,32,33,34]. At the same time, existing literature remains largely focused on surgical techniques, case selection criteria, and clinical outcomes, rather than on the structural decomposition of the maxilla as a planning tool. Important contributions have been made regarding anatomical feasibility, cortical thickness, and clinical indications for transnasal implants [9,19], yet there is still no clearly established framework that separates and analyzes the maxilla according to functionally relevant osseous components in order to guide implant planning in a reproducible way. In this context, a segmentation-based structural approach may offer a meaningful advantage. By partitioning the maxillary bone into anatomically and clinically relevant osseous components and allowing selective visualization of cortical and trabecular structures, such an approach may facilitate identification of favorable cortical support areas and more clearly delineate possible implant trajectories in transnasal and perinasal regions [7,32,33,34]. The concept of multi-component anatomical bone segmentation (MCABS) is proposed here as a novel framework for this purpose, shifting the analysis from global volumetric representation to component-based structural interpretation of the atrophic maxilla. By integrating anatomical, biomechanical, and digital-planning perspectives, this approach may contribute to a more structured and reproducible understanding of implant planning in anatomically complex maxillary cases. Furthermore, the present study represents a proof-of-concept based on a single clinical case and should be interpreted as a methodological demonstration. Within this framework, tricortical configurations should be understood as planning-oriented anatomical concepts rather than validated biomechanical endpoints. Accordingly, implant trajectories must be carefully evaluated to avoid interference with anatomically sensitive structures, particularly the nasolacrimal duct. In this context, selective cortical visualization may also contribute to safer planning by allowing more accurate assessment of implant proximity to critical structures such as the nasolacrimal duct (NLD). The aim of the present study was therefore to introduce a segmentation-assisted workflow with emphasis on evaluating safety in relation to the NLD and defining anatomical eligibility through identification of favorable cortical pathways. The concept of multi-component anatomical bone segmentation (MCABS) is proposed here as a structural tool that enables selective visualization of cortical bone and facilitates direct spatial assessment of implant trajectories in relation to critical anatomical structures, including the nasolacrimal duct (NLD), without reliance on conventional measurement methods. From this perspective, implant planning in the atrophic maxilla should not be limited to volumetric assessment but should incorporate a structured evaluation of cortical engagement patterns, including the identification of potential bicortical and, in selected cases, tricortical anchorage pathways. In this context, beyond technical feasibility, two critical aspects remain insufficiently defined: the safety of implant placement in relation to adjacent anatomical structures, particularly the nasolacrimal duct (NLD), and the anatomical eligibility of patients for such procedures.

## 2. Materials and Methods

### 2.1. Study Design and Positioning of the Manuscript

This study was developed as a methodological proof-of-concept supported by a single clinical demonstration. The primary focus was placed on the proposed workflow itself, considered the core element of innovation, rather than on the individual patient. Accordingly, this manuscript does not aim to provide a comparative, controlled, or quantitative validation but instead offers a structured presentation of the conceptual foundation of the workflow and its structured clinical implementation in a representative case. A central component of the proposed workflow is the use of automated multi-component anatomical bone segmentation performed with Ez3D-i (VATECH, Hwaseong-si, Republic of Korea), where segmentation was performed using the DAVIS Toolkit (DTKSAgentServer version 1.1.1.1 Win64) implemented via Eyes of AI software (EAI Engine version 2.0.2.0),The resulting segmented datasets were subsequently transferred into BlueSkyPlan software, where the entire downstream digital workflow was carried out, including anatomical interpretation, implant trajectory planning, and guided surgery design. This cross-platform integration enabled a continuous and coherent transition from segmentation to clinically applicable implant planning within a unified operative framework. The proof-of-concept component was included to demonstrate feasibility under real clinical conditions, including CBCT acquisition, segmentation, three-dimensional reinterpretation of the nasal and anterior maxilla anatomical structures, implant trajectory planning, surgical guide design, and guided intraoperative application. Particular emphasis was placed on the identification and utilization of alternative anchorage pathways within the nasal–paranasal complex in cases of severe maxillary atrophy. The selected case reflects a clinically relevant scenario of rehabilitation in a severely atrophic maxilla requiring non-conventional implant trajectories and extended anchorage strategies. A 57-year-old female patient with no reported systemic diseases presented for full-arch oral rehabilitation. Clinical and radiological examination revealed a severely atrophic edentulous maxilla restored with a complete removable denture. Due to the advanced degree of maxillary atrophy and the limited amount of residual bone available for conventional implant placement, the case was considered suitable for demonstrating the proposed segmentation-assisted workflow for anatomical assessment and planning of potential paranasal implant trajectories. However, no claim is made that this case is representative of the full spectrum of anatomical variability, and the use of a single case does not allow generalization regarding accuracy, reproducibility, or broader clinical applicability. All procedures were performed within routine clinical practice, following standard diagnostic protocols, with informed consent obtained for treatment and for the anonymized scientific use of patient data.

### 2.2. Imaging Acquisition

The patient completed a CBCT examination of the maxilla. The CBCT system used was a CS 8100 3D, manufactured by Carestream Dental (Carestream Dental, Atlanta, GA, USA), and CBCT images were acquired using a voxel size of 0.15 mm, a tube voltage of 90 kVp, an X-ray tube current of 3 mA, and a field of view of 8 × 8 cm. It was decided to use the 0.15 mm voxel rather than the 0.2 mm voxel in order to improve the resolution of the CBCT image as well as the quality of segmentation.

### 2.3. Software Environment and Multi-Component Segmentation

**Figure 1:**
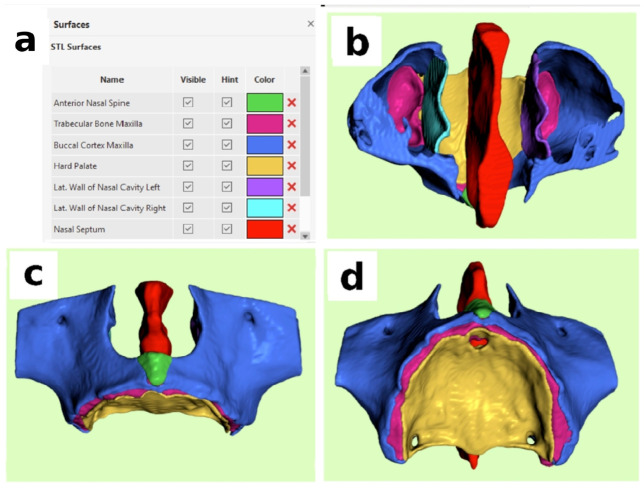
Multi-component maxillary segmentation with merged components. (**a**) Color-coded segmentation of the anatomical components, including the anterior nasal spine, trabecular bone maxilla, buccal cortex maxilla, hard palate, left and right lateral walls of the nasal cavity, and nasal septum, as detailed in the figure. (**b**) Superior view of the multicomponent assembly. (**c**) Frontal view of the multicomponent assembly. (**d**) Inferior view of the multicomponent assembly.

The CBCT volume was imported into Ez3D-i (VATECH, Hwaseong-si, Republic of Korea), where segmentation was performed using the DAVIS Toolkit (DTKSAgentServer version 1.1.1.1 Win64) implemented via Eyes of AI software (EAI Engine version 2.0.2.0), resulting in seven maxillary components—anterior nasal spine, trabecular bone maxilla, buccal cortex maxilla, hard palate, left and right lateral walls of the nasal cavity, and nasal septum—identified according to the color coding in Figure 1a, while Figure 1b–d illustrates the multicomponent assembly in superior, frontal, and inferior views, respectively, with all components visualized within the BlueSkyPlan software. The segmented anatomical structures were reviewed against the original CBCT dataset in axial, coronal, and sagittal planes by an experienced dentomaxillofacial radiologist. No manual corrections were considered necessary, as the segmented cortical surfaces showed satisfactory correspondence with the anatomical cortical boundaries visible in the original CBCT images.

**Figure 2:**
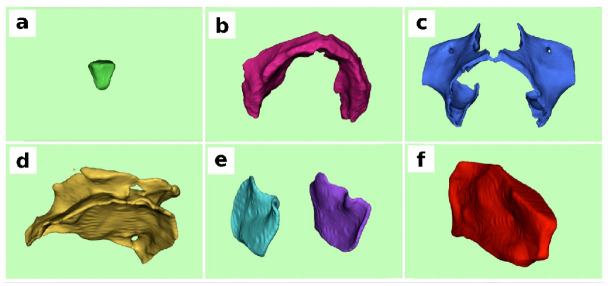
Multi-component segmentation of the maxillary bone into separate components. (**a**) Anterior nasal spine (green); (**b**) trabecular bone maxilla (magenta); (**c**) buccal cortex maxilla (blue); (**d**) hard palate (yellow); (**e**) left and right lateral walls of the nasal cavity (light blue and purple); (**f**) nasal septum (red).

The STL files generated in Ez3D-i, together with the original CBCT DICOM dataset, were imported into BlueSkyPlan software version 5.0.29 (64-bit) from Blue Sky Bio LLC, Libertyville, IL, USA, where their spatial alignment with the DICOM volume was performed automatically by the software. The multicomponent segmentation of the maxillary bone is illustrated in Figure 2, where Figure 2a–f displays the individual anatomical structures described above, collectively representing the seven components identified.

**Figure 3:**
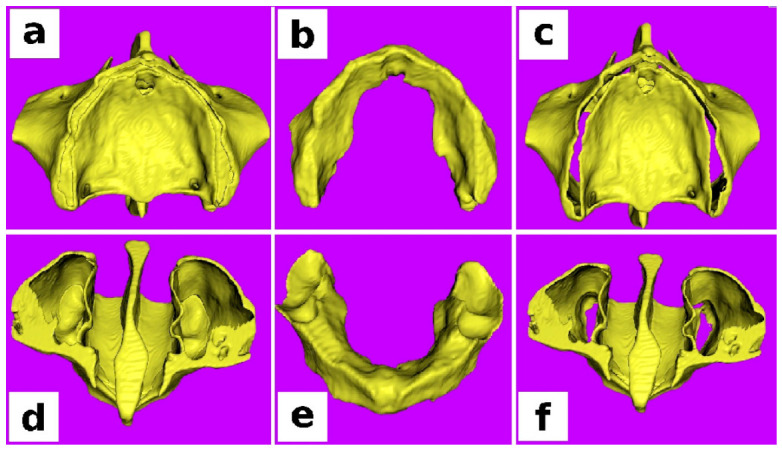
Removal of trabecular bone from the maxillary model. (**a**–**c**) Occlusal view illustrating the maxilla after exclusion of the trabecular bone. (**d**–**f**) Superior view of the maxilla following trabecular bone removal.

The BlueSkyPlan software provides an export function that allows STL files to be selectively merged in customizable combinations, enabling the exclusion of specific anatomical structures, such as trabecular bone. This approach facilitates the generation of new composite STL models, allowing targeted visualization of clinically relevant anatomical components. Thus, Figure 3 illustrates the exclusion of trabecular bone from the full maxillary model, resulting in a trabecular-free maxillary structure. In Figure 3a–c, we see the occlusal view illustrating the maxilla after exclusion of the trabecular bone, and in Figure 3d–f, the superior view of the maxilla following trabecular bone removal is presented. The resulting model highlights the cortical structures after elimination of the trabecular component.

**Figure 4:**
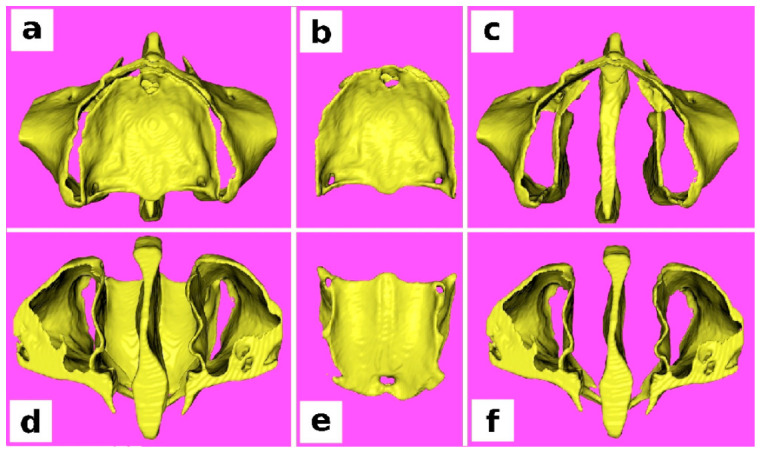
Exclusion of the hard palate from the maxillary structure, after its segmentation from the trabecular bone. (**a**–**c**) Occlusal view and (**d**–**f**) superior view.

In a manner analogous to Figure 3, Figure 4 presents the exclusion of the hard palate from the maxillary structure after its segmentation from the trabecular bone in the previous step, Figure 4a–c, the occlusal view and Figure 4d–f, the superior view.

### 2.4. Segmentation-Based Identification of Tricortical Bone in the Anterior Maxilla

**Figure 5:**
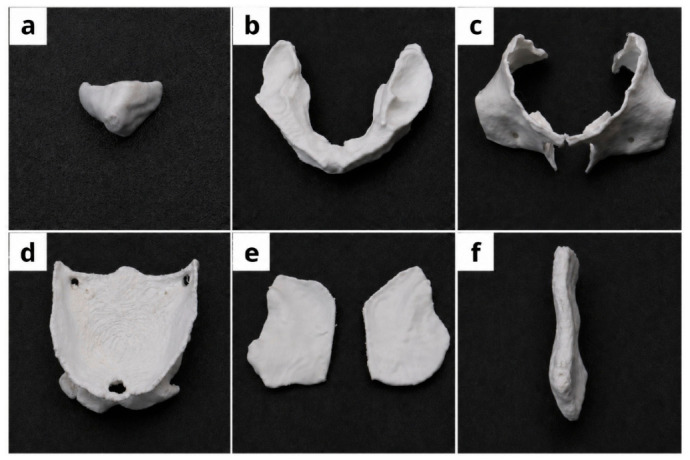
3D-printed models. (**a**) Anterior nasal spine; (**b**) trabecular bone maxilla; (**c**) buccal cortex maxilla; (**d**) hard palate; (**e**) left and right lateral walls of nasal cavity; (**f**) nasal septum.

The use of study models in medicine is increasingly associated with their physical 3D printing, enabling improved spatial orientation and direct examination of anatomical structures in a tangible format. Advances in printing technologies have substantially simplified these workflows, supporting their routine clinical implementation. In dentistry, the integration of multi-component segmentation with selective STL merging enables the generation of customized composite models through the targeted exclusion of specific anatomical structures, such as trabecular bone or the hard palate. This approach provides enhanced control over the anatomical content of the model and facilitates focused visualization of clinically relevant regions, representing a flexible alternative not only to conventional monolithic 3D models but also to purely digital representations by allowing direct, life-size assessment of anatomical structures. For this reason, our workflow incorporates the physical 3D printing of the segmented components to enable their analysis as tangible study models, as illustrated in Figure 5, where Figure 5a–f show the following 3D-printed models: anterior nasal spine, trabecular bone of the maxilla, buccal cortex of the maxilla, hard palate, left and right lateral walls of the nasal cavity, and nasal septum.

**Figure 6:**
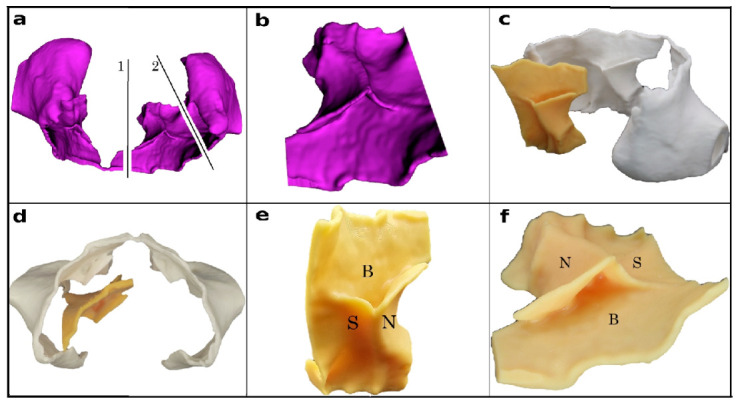
Segmentation and physical modeling of the tricortical convergence zone (TCZ) in the anterior maxilla. (**a**) The two planes (1 and 2) used to section the TCZ are illustrated. (**b**) The TCZ in digital format following sectioning. (**c**,**d**) 3D-printed study models of the TCZ together with the vestibular maxillary cortex; (**c**) posterior view and (**d**) superior view. (**e**) Anterior view of the TCZ. (**f**) Superior view TCZ. Notation represents: B for the buccal/vestibular cortex, N for the nasal wall cortex, and S for the sinus cortical wall.

To better analyze the vestibular cortex of the maxilla, we printed the study model, which we sectioned in the zone of the intersection of the lateral nasal wall cortex, the vestibular maxillary cortex, and the medial sinus wall, so that we could better analyze this tricortical convergence zone (TCZ). In Figure 6a, the two planes (1 and 2) used to section the vestibular maxillary cortex are illustrated. In Figure 6b, the TCZ is presented in digital format following sectioning. In Figure 6c,d, the TCZ is shown as a 3D-printed study model together with the vestibular maxillary cortex, enabling improved analysis for implant simulation. Figure 6c presents a posterior view of these models, while Figure 6d shows a superior view. Figure 6e illustrates an anterior view of the TCZ, whereas Figure 6f depicts a superior view of the same structure. In Figure 6b,c, the three cortical structures—namely, the vestibular (buccal) maxillary cortex, the lateral nasal wall cortex, and the medial sinus wall—are denoted as follows: B for the buccal/vestibular cortex, N for the nasal wall cortex, and S for the sinus cortical wall.

### 2.5. Description of Cutting Plane and NasoLacrimal Duct (NLD)

**Figure 7:**
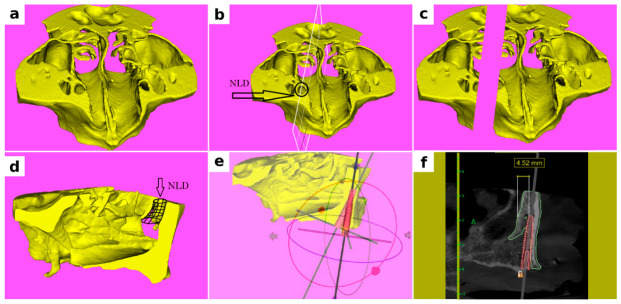
Segmentation-based visualization and implant simulation in relation to the nasolacrimal duct (NLD) in BlueSkyPlan. (**a**) Segmented study model without multi-component segmentation, showing the nasolacrimal duct. (**b**) Vertical sagittal cutting plane aligned with the trajectory of the nasolacrimal duct and passing through its midpoint. (**c**) Sectioning of the maxillary model and removal of the distal portion. (**d**) The remaining maxillary segment has improved visualization of the nasolacrimal duct. (**e**) Implant simulation within the section plane, showing its relationship to the nasolacrimal duct and the buccal cortex of the maxilla. (**f**) Measurement from the implant margin to the nasolacrimal duct.

To describe the position of the simulated implant relative to the nasolacrimal duct (NLD), Figure 7a is based on the segmented study model in BlueSkyPlan without applying multi-component segmentation, as this enables the visualization of anatomical structures that are not captured by such multi-component segmentation, such as the nasolacrimal duct. The spatial description of the cutting plane from Figure 7b was oriented along the vertical sagittal axis, in alignment with the trajectory of the nasolacrimal duct, and should intersect its midpoint. To ensure that the distance to the buccal cortical plate of the nasolacrimal duct is as accurate as possible, the section plane must satisfy an additional condition: it should be positioned and oriented to capture the minimal distance to the anterior aspect of the maxillary cortex, thereby approximating the true anatomical distance in the context of implant simulation. After removing the distal portion of the maxillary model following the sectioning performed in Figure 7c using the plane described above, the remaining maxillary segment provides an improved visualization of the nasolacrimal duct (Figure 7d). Within the section plane obtained after cutting, as illustrated in Figure 7e, implant simulation can be performed while ensuring safe distances from adjacent structures, namely the nasolacrimal duct (NLD) and the buccal cortical plate of the maxilla. At this stage of the workflow, the implant model can be selected according to the manufacturer-specific dimensions—namely, length and diameter—to ensure safe placement. This represents a critical clinical decision point, as it directly determines the patient’s eligibility for treatment with an implant placed in this anatomical region. For the proof-of-concept application, a pterygoid implant from the BIO|Pterygoid category (Blue Sky Bio LLC, Libertyville, IL, USA), type IPJN3722, was selected. The implant had an apical diameter of 2.31 mm, an occlusal diameter of 3.70 mm, and a length of 22.50 mm. In Figure 7f, a measurement was performed within the previously described section plane, from the implant margin to the nasolacrimal duct, yielding a distance of 4.52mm. The nasolacrimal duct (NLD) was identified directly within the CBCT dataset using multiplanar visualization and three-dimensional anatomical correlation. The reported distance of 4.52 mm corresponds to the minimum distance measured within the section plane described in the manuscript and used for model sectioning. More specifically, this value represents the shortest distance between the posterior surface of the planned implant and the visible boundary of the nasolacrimal duct within that assessment plane. This measurement was intended to illustrate the spatial relationship between the planned implant trajectory and the NLD within the proposed workflow rather than to establish a generalized safety threshold. 

### 2.6. Implant Placement in the Study Model Using a Surgical Guide

**Figure 8:**
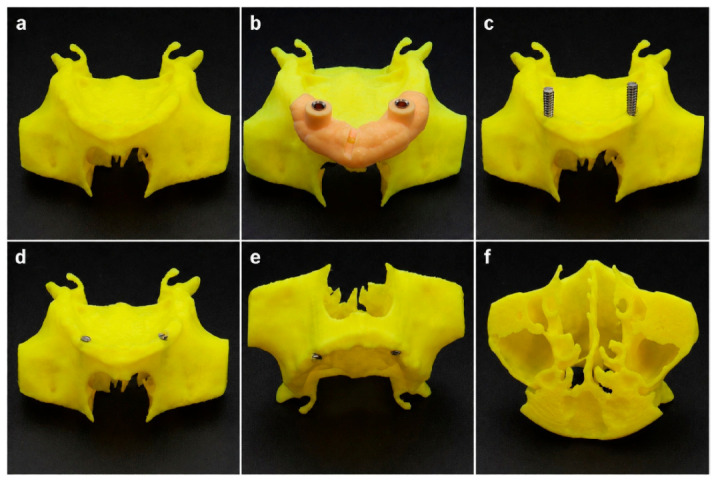
Surgical guide-assisted implant insertion in the study model using BlueSkyPlan. (**a**) Segmented maxillary model used for guide design. (**b**) Surgical guide superimposed on the study model. (**c**) Partial implant insertion following guided pilot drilling. (**d**–**f**) Model with fully inserted implants: occlusal view (**d**), anterior view (**e**), and superior view (**f**).

Based on the segmented maxillary model in BlueSkyPlan (Figure 8a), and using the implant insertion protocol described above, a surgical guide was generated, as shown in Figure 8b, superimposed on the study model within the BlueSkyPlan environment. The surgical guide used a StecoGuide system (Steco-System-Technik, Hamburg, Germany) and a titanium outer sleeve (REF M.27.02.D350L5; outer diameter 4 mm, inner diameter 3.5 mm, length 6.0 mm). The pilot drill was performed using a 2 mm drill guided through an inner sleeve positioned within an outer sleeve with an outer diameter of 3.5 mm and an inner diameter of 2.0 mm (STECO, model M.27.03.D200L6). The first guide hole was prepared using a 2 mm pilot drill through the inner sleeve positioned within the outer sleeve; subsequently, the inner sleeve was removed, and the second drilling step was performed using a 3.5 mm drill guided through the outer sleeve. In Figure 8c, following drilling through the surgical guide, the implants are shown partially inserted. Figure 8d–f presents successive views of the model with the implants fully inserted: occlusal view (d), anterior view (e), and superior view (f), with no evidence of implant perforation beyond the boundaries of the model.

## 3. Results

### 3.1. Workflow Feasibility

**Figure 9:**
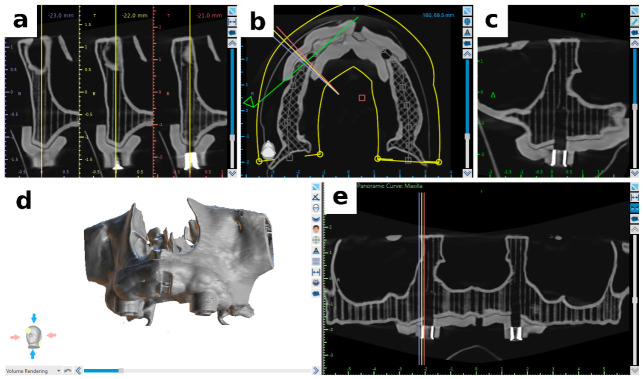
Verification of surgical guide seating on the study model. (**a**) Cross-sectional view of the guide and study model. (**b**) Axial section. (**c**) Mesiodistal section. (**d**) 3D rendered view of the guide positioned on the model, showing the safety verification window and the visible superior crest margin. (**e**) Panoramic section demonstrating the accurate adaptation between the guide and the study model.

This verification of workflow was performed using CBCT volumetric acquisition of the study model together with the surgical guide, initially to assess the accuracy of guide seating on the model and subsequently to evaluate the correctness of implant insertion. A fundamental requirement for accurate implant placement using surgical guides is the precise seating of the guide on the operative field. In Figure 9a, a cross-sectional view of the guide and the study model is presented. Figure 9b shows an axial section, while Figure 9c illustrates a mesiodistal section of the same components. Figure 9d depicts a 3D rendered view of the study model with the guide in position, including the safety verification window created in the guide, through which the superior margin of the crest is visible. Figure 9e presents a panoramic section through the guide and the study model, clearly demonstrating the accurate adaptation between the guide and the model.

### 3.2. CBCT-Based Radiological Verification

The verification of implant insertion in the study model, based on the planning performed on the patient’s CBCT, relied on an approach designed to overcome the artifacts generated by metallic implants inserted into the model. Due to these artifacts, reliable linear measurements could not be performed; therefore, the assessment of accuracy was based on the superimposition of implant axes.

This method involved the generation of custom implants within the study model containing the inserted metallic implants. Specifically, virtual implants with a length of 50 mm and a diameter of 1 mm were created and positioned along the central axes of the metallic implants. This assembly of custom implants was exported as an STL file from BlueSkyPlan, subsequently reimported, and superimposed onto the patient’s CBCT dataset. The qualitative assessment was based on the superimposition of the virtual implant axes and the custom-created implant axes used for verification. The custom verification axes were generated with a diameter of 1 mm and were manually superimposed onto the corresponding virtual implant axes within the CBCT environment. The superimposition procedure was performed by aligning the two CBCT datasets and subsequently matching the corresponding implant axes. As illustrated in the figure, the two axes could not be visually distinguished from one another along their entire length (50 mm), indicating that any deviation between them was smaller than the diameter of the verification axis itself. Based on this observation, the discrepancy was considered to be less than 1 mm.

We emphasize that this assessment was intended as a qualitative verification of workflow feasibility and internal consistency rather than as a formal quantitative accuracy analysis. Precise submillimetric measurements were considered unreliable in this setting because of CBCT-related artifacts and image limitations associated with metallic implant visualization.

**Figure 10:**
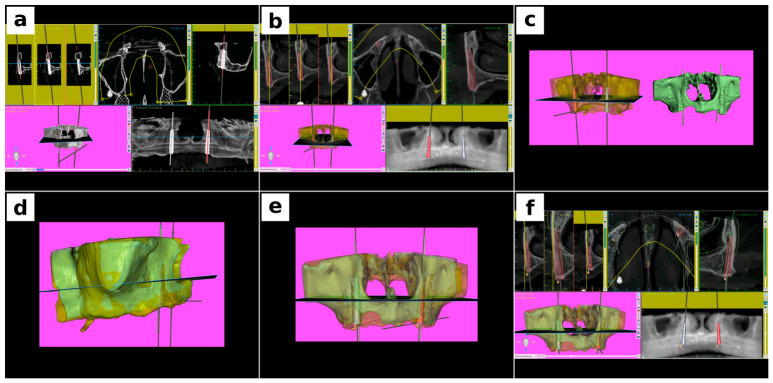
Axis-based verification of implant insertion by superimposition. (**a**) Study model with metallic implants inserted using the surgical guide. (**b**) Patient CBCT with virtually planned implants and their axes. (**c**) Side-by-side visualization of the study model, including the custom axial implants, and the patient’s CBCT dataset prior to alignment. (**d**) Superimposition of the study model onto the CBCT dataset in the lateral view. (**e**) Superimposition in frontal view. (**f**) Superimposition in sectional views, demonstrating minimal deviation between the planned implant axes and the corresponding custom implants.

The workflow is illustrated in Figure 10. (a) Study model with metallic implants inserted using the surgical guide. (b) Patient CBCT with virtually planned implants and their corresponding axes. (c) Combined visualization of the CBCT dataset and the study model with the custom implants. (d) Superimposition of the two datasets in lateral view. (e) Superimposition in frontal view. (f) Superimposition in sectional views.

As demonstrated in Figure 10d–f, the discrepancies between the planned implant axes and the custom implants aligned within the study model are negligible, remaining below 1 mm.

### 3.3. Volumetric Verification of Apical Implant Positioning Within the Tricortical Bone Zone (TCZ) in the Study Model

**Figure 11:**
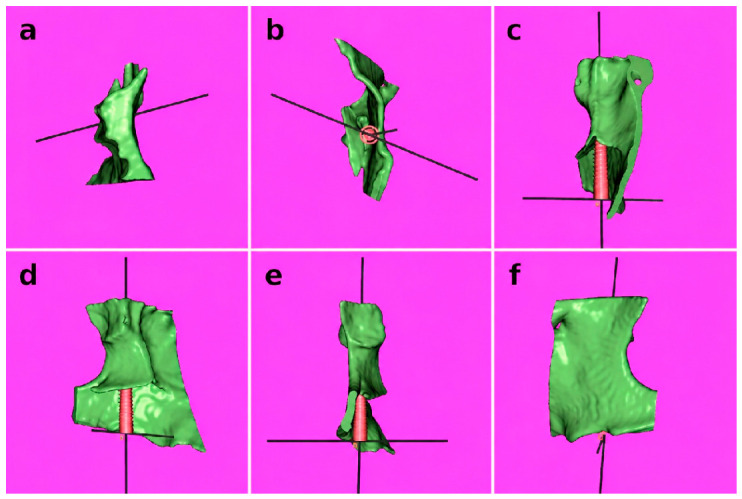
Volumetric verification of apical implant positioning within the tricortical bone zone (TCZ). (**a**) Superior view. (**b**) Occlusal view. (**c**) Distal view. (**d**) Anterior view. (**e**) Mesial view. (**f**) Frontal view. The implant apex is fully contained within the TCZ in all perspectives, with no evidence of perforation beyond its boundaries.

Volumetric verification of apical implant positioning within the tricortical bone zone (TCZ) was performed using a six-view assessment of the study model (Figure 11a–f). The TCZ and the inserted implant were evaluated from superior (Figure 11a), occlusal (Figure 11b), distal (Figure 11c), anterior (Figure 11d), mesial (Figure 11e), and frontal (Figure 11f) perspectives. Across all views, the implant apex remained fully contained within the TCZ, with no evidence of perforation beyond its boundaries. This step represents a practical volumetric verification, analogous to direct inspection on a printed study model, confirming that no portion of the implant emerges outside the TCZ.

## 4. Discussion

The present proof-of-concept study proposes a segmentation-assisted workflow based on multi-component anatomical bone segmentation (MCABS) for the anatomical evaluation of potential paranasal implant trajectories in the severely atrophic maxilla. Rather than treating the maxilla as a single undifferentiated osseous volume, the workflow was designed to facilitate selective visualization of anatomically relevant cortical structures and to support identification of potential implant pathways in anatomically complex regions. A key methodological aspect of the proposed workflow was the use of axis-based verification. Direct linear measurements were considered unreliable because metallic implants inserted into the study model generated CBCT artifacts capable of affecting boundary definition and geometric interpretation. For this reason, superimposition of planned and executed implant axes was adopted as the primary verification strategy. Within the limitations of a proof-of-concept design, this approach provided a practical method for assessing workflow consistency and technical feasibility. The novelty of the present study does not reside in CBCT-based planning, anatomical segmentation, transnasal implants, or multicortical anchorage individually, as all of these concepts have previously been described [6,7,8,9,10,11,12,13,16,17,18,19,20]. Rather, the proposed contribution lies in the integration of multi-component anatomical bone segmentation into a structured workflow designed to facilitate selective cortical visualization, direct assessment of the nasolacrimal duct (NLD), and evaluation of anatomical eligibility for potential paranasal implant trajectories in severely atrophic maxillae. One conceptual implication of this approach is the shift from a predominantly volumetric interpretation of bone availability toward a structural interpretation focused on cortical anatomy. Previous investigations have emphasized that the mechanical value of residual maxillary bone depends not only on bone volume but also on the location, continuity, and accessibility of cortical structures capable of providing implant anchorage [6,7,10]. Jensen et al. highlighted the importance of the paranasal region, particularly the lateral pyriform rim and M-point area, as strategic anatomical sites for implant support in severely atrophic maxillae [35]. Similarly, Calin et al. demonstrated that specific regions of the anterior maxilla may provide greater cortical thickness than adjacent anatomical areas, reinforcing the concept that local cortical anatomy may be more informative than overall residual bone volume alone [7]. Within this context, MCABS may facilitate identification of structurally relevant cortical pathways that may be less apparent in conventional whole-volume representations. A particular feature of the proposed workflow is the direct visualization of the spatial relationship between the planned implant trajectory and the nasolacrimal duct. Previous authors have emphasized the importance of careful anatomical assessment when planning transnasal or paranasal implants because of the proximity of critical anatomical structures, including the NLD [9,17]. In the present workflow, the segmented anatomical model enabled three-dimensional assessment of the implant pathway relative to the duct throughout its course, thereby facilitating individualized anatomical evaluation. Nevertheless, the present study does not establish safety thresholds for NLD preservation and should be interpreted as an anatomical planning demonstration rather than a validation of surgical safety. No evidence-based minimum safety distance currently exists for this indication, and establishing such thresholds was beyond the scope of the present proof-of-concept study. The findings also support the broader concept that transnasal and paranasal implant trajectories may represent strategically relevant cortical anchorage pathways in carefully selected patients. Previous clinical reports have suggested that such implants may complement established graftless rehabilitation concepts and improve implant distribution in severely atrophic maxillae [4,8,17,18,19,20]. Nicoli et al. described extra-long implants directed toward the nasal wall as a feasible alternative in complex full-arch rehabilitation [4], while Nunes et al. reported the integration of transnasal implants within zygomatic implant-supported rehabilitation concepts [17]. Gelpi et al. subsequently reported favorable outcomes for nasal and transnasal implants placed in severely atrophic anterior maxillary sites [19]. Collectively, these reports support the clinical relevance of the anatomical regions explored in the present study. Another important aspect concerns the concept of tricortical anatomical engagement. This concept should be interpreted as an anatomical planning observation rather than a validated biomechanical phenomenon. The present study does not demonstrate superior implant stability, insertion torque, stress distribution, or clinical performance associated with tricortical engagement. Instead, it describes a specific anatomical configuration involving the lateral nasal wall cortex, vestibular maxillary cortex, and medial sinus wall that may be visualized through segmentation-assisted planning. Although bicortical anchorage has been widely associated with improved primary stability [10,28,36], the potential biomechanical significance of tricortical anatomical configurations remains speculative and requires dedicated biomechanical, radiological, and clinical investigation. Consequently, tricortical anatomical engagement should be regarded as a planning-oriented anatomical concept rather than a validated biomechanical endpoint. The present workflow may also have relevance for guided implantology in anatomically complex regions. The combination of segmentation-assisted planning, three-dimensional modelling, and guided insertion enabled the consistent transfer of the planned trajectory to the study model. Although this does not constitute clinical validation, it supports the technical feasibility of integrating segmentation-based planning with guided surgical workflows. In anatomically demanding regions characterized by limited visibility, restricted access, and proximity to critical structures, guided approaches may contribute to improved control of implant trajectory and placement accuracy [37,38]. Several limitations should be acknowledged. First, the study was based on a single clinical case and therefore does not permit conclusions regarding reproducibility, effectiveness, or broader clinical applicability. Second, quantitative validation of segmentation accuracy, interobserver agreement, and reproducibility was beyond the scope of the present proof-of-concept study. Third, no biomechanical validation, finite element analysis, or clinical outcome assessment was performed. Fourth, no comparison with alternative segmentation platforms was undertaken, and therefore no conclusions regarding superiority or comparative performance can be made. Finally, no safety thresholds for the nasolacrimal duct can be proposed based on a single anatomical demonstration. Within these limitations, the proposed workflow may contribute to a more structured anatomical interpretation of the severely atrophic maxilla. By facilitating selective cortical visualization and direct assessment of the relationship between planned implant pathways and adjacent anatomical structures, MCABS may support preoperative anatomical evaluation and future development of segmentation-assisted planning strategies for complex implant rehabilitation. Future studies should focus on anatomical validation, reproducibility assessment, biomechanical investigation, and clinical outcome analysis before broader clinical adoption can be considered.

## 5. Conclusions

The present proof-of-concept study describes a segmentation-assisted workflow based on multi-component anatomical bone segmentation (MCABS) for the anatomical evaluation of potential paranasal implant trajectories in the severely atrophic maxilla. The proposed approach facilitates selective visualization of cortical structures and direct assessment of the spatial relationship between planned implant pathways and adjacent anatomical structures, including the nasolacrimal duct.

Within the proposed workflow, cortical pathway-oriented planning is presented as an anatomical interpretation framework intended to support evaluation of potential implant trajectories in anatomically complex regions. In selected cases, segmentation-assisted planning may also reveal anatomical configurations involving multiple cortical boundaries, including potential tricortical anatomical engagement. However, such observations should be regarded as anatomical planning concepts rather than validated biomechanical phenomena.

The present study should be interpreted as a methodological demonstration rather than a validated clinical protocol. No conclusions can be drawn regarding clinical superiority, reproducibility, biomechanical performance, or long-term treatment outcomes. Similarly, the workflow does not establish safety thresholds for nasolacrimal duct preservation or definitive criteria for patient eligibility.

Within the limitations of a single-case proof-of-concept design, the proposed workflow may contribute to preoperative anatomical assessment and to a more structured interpretation of cortical anatomy in the severely atrophic maxilla. Further anatomical, reproducibility, biomechanical, and clinical studies are required before broader clinical adoption can be considered.

## Data Availability

Because the study includes patient-derived imaging data, the full datasets are not publicly available for privacy and ethical reasons. De-identified methodological details related to the workflow may be made available from the corresponding authors upon reasonable request, subject to ethical and legal constraints.
